# A comparison of videolaryngoscopes for tracheal intubation in predicted difficult airway: a feasibility study

**DOI:** 10.1186/s12871-017-0318-2

**Published:** 2017-02-20

**Authors:** Maria Vargas, Antonio Pastore, Fulvio Aloj, John G. Laffey, Giuseppe Servillo

**Affiliations:** 1 Section of Anesthesia and Intensive care, Department of Neurosciences, Reproductive and Odontostomatological Sciences, University of Naples “Federico II”, Via Pansini 16, Naples, Italy; 2 Section of Anesthesia and Intensive care, Anesthesia and Intensive Care Unit, IRCCS Neuromed, Pozzilli, IS Italy; 3 Section of Anesthesia and Intensive care, Department of Anesthesia, Critical Illness and Injury Research Centre, Keenan Research Centre for Biomedical Science, St Michael’s Hospital, University of Toronto, Toronto, Canada

**Keywords:** Videolaryngoscopes, Predicted difficult intubation, Intubation difficulty scale, Imago V-blade, Glidescope

## Abstract

**Background:**

Videolaryngoscopy has become increasingly attractive for the routine management of the difficult airway. Glidescope® is well studied in the literature while imago V-Blade® is a recent videolaryngoscope. This is a feasibility study with 1:1 case-control sequential allocation comparing Imago V-Blade ® and Glidescope® in predicted difficult airway settings.

**Methods:**

Two senior anesthesiologists with no clinical experience in video assisted intubation but previously trained in a simulated scenario, performed the endotracheal intubations with Imago V-Blade® and Glidescope®. A third experienced anesthesiologist supervised the procedures. Forty-two patients, 21 for each group, with the presence of predicted difficult airway according to the Italian guideline were included. The primary end point is the feasibility of intubation. The secondary end-points are the success to intubate in the first attempt, the intubation time, the Cormack and Lehane score view, the comparison of the intubation difficulty scale (IDS) score and the need for maneuvers to aid the endotracheal intubation comparing Imago V-Blade® and Glidescope®.

**Results:**

The intubation was achieved in 100% of cases in both groups. No differences were found in the first-attempt success rate (*p* = 0.383), intubation time (*p* = 0.280), Cormack and Lehane score view (*p* = 0.799) and IDS score (*p* = 0.252). Statistical differences were found in external laryngeal pressure (*p* = 0.005), advancement of the blade (*p* = 0.024) and use of increasing lifting force (*p* = 0.048).

**Conclusions:**

This feasibility study showed that the intubation with the newly introduced Imago V-Blade® is feasible. Further randomized and/or non-inferiority trials are needed to evaluate the benefit of Imago V-Blade® in this procedure.

**Trial registration:**

Clinicaltrials.gov NCT02897518. Retrospectively registered 25 August 2016

## Background

In recent years, videolaryngoscopy has become increasingly attractive for the routine management of the difficult airway. Videolaryngoscopes offer several advantages during endotracheal intubation. The Glidescope® is a videolaryngoscope for indirect laryngoscopy significantly different from Macintosh because of its rigid and 60° angled blade. The view of the glottis provided by the Glidescope® seems to be improved compared with the Macintosh laryngoscope in difficult airways [1]. During difficult intubations, the Glidescope® has been associated with more successful endotracheal intubation compared with the C-MAC® videolaryngoscope [2].

**Fig. 1:**
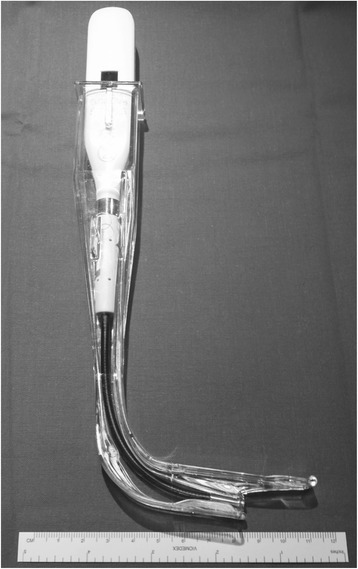
Imago V-Blade® 90° disposable blade with integrated channel for endotracheal tube. The shape of the blade is perpendicular to the main device axis

The Imago V-Blade® (Fig. 1) is a recent videolaryngoscope equipped with a wireless video-assisted stylet within it’s 90° angled blade. Both the Glidescope® and Imago V-Blade® have a digital camera at the tip of the blade extending the view angle beyond that of a standard Macintosh laryngoscope. The Imago V-Blade® has a channel for the tracheal tube to be preloaded before the laryngoscopy. This is the first feasibility study comparing the use of the Imago V-Blade® with the Glidescope® in predicted difficult endotracheal intubation performed by non experienced anesthesiologist. The primary end point is the feasibility of intubation. The secondary end-points are the success to intubate in the first attempt, the intubation time, the Cormack and Lehane score view, the comparison of the intubation difficulty scale (IDS) score and the need for maneuvers to aid the endotracheal intubation comparing Imago V-Blade ® and Glidescope®.

## Methods

### Study design and patient selection

This is a feasibility study approved by the ethics committee of University of Naples “Federico II” (protocol number 123/15) and registered in clinical trial (Trial registration NCT02897518). All patients provided a written informed consent for study participation. Patients admitted to the operation rooms of University of Naples “Federico II” and requiring endotracheal intubation for general anesthesia were consecutively screened for the presence of predicted difficult airway according Italian guideline [3]. According to this guideline, the presence of one or more of the following parameters may be considered highly predictive of difficult intubation: Mallampati class 3–4, inter-incisor distance < 30 mm, mental-thyroidal distance < 60 mm, large prominence of superior incisors above inferior incisors uncorrectable with jaw-thrust, reduced head and neck motility, and reduced mental-jugular distance. Patients matching more than 1 of the previous criteria stated by the current Italian guideline were included in this case-controlled study. Patients were sequential allocated with a 1:1 ratio to each device/group. The case group received endotracheal intubation with the Imago V-Blade® and the control group underwent tracheal intubation with the Glidescope®.

Patients 1) without criteria for predicted difficult airway; 2) those requiring emergency surgery; 3) aged < 18 years; or 4) declined consent to participate, were excluded from this study.

During the reviewing process the primary end-point has been changed. The authors originally designed this study as non–inferiority study and then the primary end point was a comparison of the IDS score. However, since this is the first study evaluating a new device, we found more correct to call this study as feasibility study. As a consequence, the primary end-point is the feasibility of intubation with the new device Imago V-Blade®.

### End points

The primary end point is the feasibility of intubation. The secondary end-points are the success to intubate in the first attempt, the intubation time, the Cormack and Lehane score view, the comparison of the intubation difficulty scale (IDS) score and the need for maneuvers to aid the endotracheal intubation comparing Imago V-Blade ® and Glidescope®.

### Technical aspects

Non-invasive blood pressure, electrocardiogram and pulse-oximetry were normally monitored for each patient. Patients were preoxygenated for 5 min with 100% oxygen. General anesthesia was induced with a standardized regimen that included intravenous fentanyl (2 μg/kg) and propofol (2 mg/kg). When the patient lost consciousness, bispectral index < 60, rocuronium (0.8 mg/kg) was administered. A peripheral nerve stimulator (TOF-Watch® Organon, Dublin, Ireland) was used to confirm that the train of- four ratio decreased to zero, which indicated an ideal intubation condition had been achieved. Mask ventilation with 100% oxygen was delivered to all patients during induction.

All intubations were performed by two senior anesthesiologists (A and B) with 10 years of experience in conventional endotracheal intubation but without experience in video assisted intubation with Imago V-Blade® or the Glidescope®. Anesthesiologist A performed all intubations with the Imago V-Blade® (group I) while anesthesiologist B used the Glidescope® (group G). A third anesthesiologist (C) with experience in video assisted intubation with both devices was present in the operation room. If the anesthesiologist A or B failed intubation after 2 attempts, anesthesiologist C took over and completed the maneuver. Otherwise, after 3 attempts the patient was awakened and intubated via a fiberoptic bronchoscopy. Furthermore, the operating room was equipped by devices recommended by Italian guidelines for airway control and difficult airway management [3].

The successful intubation on the first attempt was defined as the tracheal tube placement with a single blade insertion. The successful intubation was confirmed by capnography and auscultation of lungs and stomach. The removal of the laryngoscope from the mouth and further manipulation of the laryngoscope inside the mouth also constituted an intubation failure. The intubation time was defined as the time period between the laryngoscopes passing the patient’ s lips and the completion of a successful intubation. The Cormack and Lehane score [3] view was reported by both the laryngoscopists as the first own observation on the video screen just after the positioning of the videolaryngoscopes and without external tracheal maneuvers. The Intubation Difficulty Score (IDS) is a validated numerical description of the difficulty of intubation based on seven quantitative and qualitative aspects of the procedure, value 0 corresponding to ideal intubation conditions, values 1–5 to slight difficulty, and values >5 to moderate to severe difficulty [4]. Maneuvers to aid the endotracheal intubations as readjusting patient’s head, external laryngeal pressure, advancement or withdrawal the blade and increased lifting force, were collected by an independent data recorder observing the procedure.

### Description of device included in the study: Imago V-Blade® and Glidescope®

**Fig. 2:**
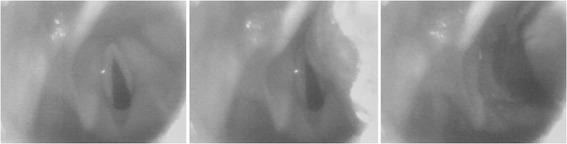
Laryngeal view from the Imago V-Blade® used in this study. The *left panel* show the glottis view with the tip of the blade inserted into the vallecular. The *middle panel* shows the placement of the endotracheal tube in front of the vocal cords with the tip of the blade slightly elevating the epiglottis. The *right panel* shows the passage of the endotracheal tube though the vocal cords keeping the tip of the blade into the vallecula

The Imago V-Blade® was equipped with a wireless video assisted stylet within the 90° angled disposable blade. Endotracheal intubation with Imago V-Blade® did not require a rigid stylet because it has a designed channel on the right for placement of the tracheal tube. This videolaryngoscope is inserted into the mouth in the midline, without displacing the tongue laterally, and advanced slowly until the epiglottis comes into view. The tip of the blade is then positioned in to the vallecula indirectly elevating the epiglottis for vocal cords exposure (Fig. 2). It is important to place the glottic opening in the centre of the monitor.

The Glidescope® is a rigid video-laryngoscope with a 60° angled blade connected by cable to a monitor. The tracheal tube used with the Glidescope® was pre-loaded with the manufacturer’s pre-configured stylet because the Glidescope® does not have a tracheal tube channel. The Glidescope® is introduced into the middle of the oral cavity, without tongue displacement, gliding along the palate and the posterior pharynx until their tip is inserted into the vallecula or posterior to the epiglottis, if the epiglottis obscures the glottis.

Anesthesiologists A and B, with 10 years of experience in conventional endotracheal intubation but without experience in video assisted intubation, were given didactic instruction on the proper use of the Imago V-Blade® and Glidescope®. As training, anesthesiologists A and B each performed 60 intubations with the assigned videolaryngoscope in a manikin with three difficult airway scenarios: 20 intubations in a normal manikin without modifications, 20 intubations in a manikin with the tongue insufflated with 110 ml of air, 20 intubations in a manikin with cervical immobilization. The anesthesiologist C, with more than 100 clinical intubations with both devices, supervised the training.

### Statistical analysis

Data are reported as means and standard deviations (± SD), proportions or median and range interquartiles (IQR) as appropriate. Normal distribution was evaluated with the Shapiro-Wilk normality test. Comparisons between groups were performed with one-way ANOVA for continuous variables. Statistical significance (p) was set at 0.05. Statistical analysis was obtained with SPSS (version 20.0, IBM®, USA). A statistical post-hoc power analysis on observed effect with probability level (α) set at 0.05 was performed to assess the power of this study. The sample size has been not calculated.

## Results

**Fig. 3:**
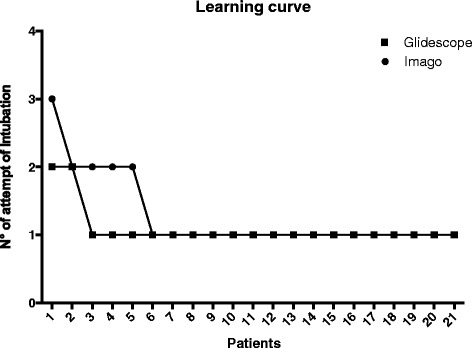
Attempts of endotracheal intubation using the Imago V-Blade® and Glidescope® groups

**Fig. 4:**
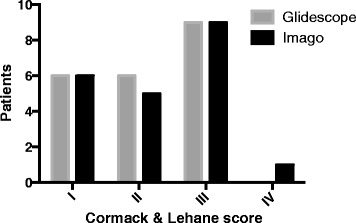
Cormack and Lehane score view for patients included in the Imago V-Blade® (*black bars*) and Glidescope® (*grey bars*) groups

Forty-two patients, 21 for each group, with the presence of predicted difficult airway according Italian guideline [3] were included in this study. Table 1 reported the main characteristics of included patients. The intubation was achieved in all patients (21/21) in the group I and group G. The intubation success rate on the first attempt between Group I and Group G was similar (Fig. 3) (*p* = 0.383). In the group I, 1/21 patient was intubated after the third attempt and 4/21 patients were intubated on the second attempt. In group G, 2/21 patients were intubated on the second third attempt of endotracheal intubation. In the group I, the median time of endotracheal intubation was 23.10 (±5.56) seconds while in the group G 25.57 (±8.75) seconds without statistical significance (*p* = 0.280). The Cormack and Lehane scores view with the two different videolaryngoscopes were not different (Fig. 4) (C-L I/II/III/IV: group I – 6/5/9/1; group G – 6/6/9/0; *p* = 0.799).

**Table 1:** Main characteristics of included patients

	Glidescope (21)	Imago (21)	*p*
Age (mean ± SD)	62 ± 10	58 ± 15	0.636
Gender (m/f)	13/8	10/11	0.352
BMI > 35	38.4 ± 2.3	38.6 ± 1.52	0.875
Mallampati Class:			0.635
I	1 (4.8%)	0	
II	5 (23.8%)	5 (23.8%)	
III	13 (61.8%)	12 (57.1%)	
IV	2 (9.5%)	4 (19%)	
Previous difficult intubation	2 (9.5%)	4 (19%)	1
Inter-incisor gap ≤3 cm	6 (28.6%)	6 (28.6%)	1
Thyromental distance <6.5 cm	6 (28.6%)	6 (28.6%)	1
Reduced jugular-mental distance	5 (23.8%)	4 (19%)	1
No possibility of maxillary prognatism	3 (14.3%)	1 (4,8%)	1
Head and neck movement <90°	3 (14.3%)	4 (19%)	1
Complete missing teeth	4 (19%)	5 (23.8%)	1
Macroglossia	4 (19%)	3 (14,3%)	1
Thyroid goiter	4 (19%)	2 (9.5%)	1
Tracheal deviation	4 (19%)	5 (23.8%)	1

**Fig. 5:**
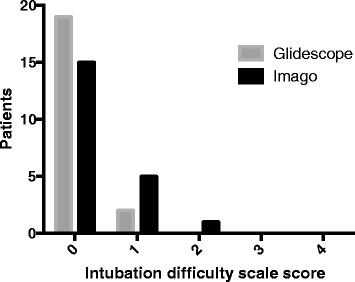
Intubation difficulty scale score for patients included in the Imago V-Blade® (*black bars*) and Glidescope® (*grey bars*) groups. IDS =0/<5/>5: group I – 15/6/0; group G – 19/2/0; *p* = 0.252)

The IDS score was less than 5 points in both groups (Fig. 5) (median and IQR for IDS: group G 1 (0-1), group I 1 (0–2) (*p* = 0.252).

Table 2 reports the maneuvers needed to aid intubation of included patients. There was no difference between devices in the need to readjust the patient’s head. Glidescope® required more external laryngeal pressure than Imago V-Blade® (group G 13/21, group I 4/21, *p* = 0.004). Advancement of the blade was more common in the group I than the group G (group I 11/21, group G 4/21, *p* = 0.024). The use of increasing lifting force more common in the group G (increasing lifting force: group G 10121, group I 4/21, *p* = 0.024).

**Table 2:** Maneuvers to aid intubation in both groups

	Glidescope (21)	Imago (21)	*p*
Readjust patient’s head	0	0	–
External laryngeal pressure	13	4	0.004
Advance blade	4	11	0.024
Withdraw blade	8	4	0.179
Increase lifting force	11	4	0.024

## Discussion

To our knowledge, this is the first feasibility study comparing Imago V-Blade® and Glidescope® for patients with a predicted difficult airway. In this study we found that intubation was feasible with Imago V-Blade® in all recruited patients. Furthermore, we found that Imago V-Blade® and Glidescope®: 1) had a high intubation success rate on the first attempt, 2) a rapid time needed for endotracheal intubation, 3) Cormack and Lehane score view in most cases less than four and 4) needed different maneuvers to aid a successful endotracheal intubation. Imago V-Blade® is a videolaryngoscope recently developed. This current prospective case-control study was designed to evaluate the performance of a new videolaryngoscopes, the Imago V-Blade® and Glidescope® since the Glidescope® is the most extensively evaluated and used videolaryngoscope.

According to the previous literature on videolaryngoscopes with 90° blade, Imago V-Blade® and Glidescope® had a high success rate and a rapid time of intubation although the blades were differently angled (90° vs 60°) [5–7]. Imago V-Blade® has a 90° blade with an integrated tube channel and the shape of the blade is perpendicular to the main device axis [8]. As similar devices, the light and view axis of Imago V-Blade® could be optically manipulated approximately 270° [9]. Glidescope® has a 60°angled blade that does not require an alignment of the oral, pharyngeal and tracheal axes [10]. The light coming from the blade of the Glidescope® offers a visual axis of approximately 270° to 300° [9]. However, the intubation rate at first pass was reached after 5 patients in the Imago V-Blade® group and after 2 patients in the Glidescope® group. Probably different device designs may result in different intubation success rate and then in a faster learning curve.

Surprisingly in this study we found 9/21 patients in each group with grade 3 of Cormack and Lehane score. Despite these data suggesting a difficult endotracheal intubation, this one was successfully reached at first attempt in all but 6 patients. These are interesting data. Firstly, the Cormack and Lehane classification simply describe a view of the glottis and not the difficulty of the tube passage during the videolaryngoscopy [11]. Secondly, although a concerning grade of Cormack and Lehane score, in this study the IDS score showed an ideal condition of intubation in more than 70% of included patients. Probably the IDS score, as a descriptive method to assess difficult endotracheal intubation, may be more appropriate than Cormack and Lehane score to predict a difficult intubation during videolaryngoscopy [11]. Thirdly, the use of additional maneuvers to aid the intubation may facilitate the tube passage during videolaryngoscopy [8]. In line with current literature, Glidescope® needed more laryngeal pressure and increasing lifting force to successfully achieve endotracheal intubations [8, 12–14], while Imago V-Blade® needed to be properly positioned at the center of the mouth. The technique for a successful endotracheal intubation whit Imago V-Blade® is to position the camera at the center of the glottis with the tip of blade inserted into the vallecula indirectly elevating the epiglottis for laryngeal exposure.

## Limitations

This study has some limitations. This is a feasibility study designed as prospective case-control study not as a randomized one. So, according to a post-hoc power analysis, this study is underpowered. The power analysis performed on observed effect size of successful intubation with 21 patients/group reached a power of 0.2. We need 127 patients for each group to reach a power of 0.8. A randomized controlled trial may add further and complete information on this topic. In this study, two trained anesthesiologists performed the endotracheal intubation with the assigned device in order to evaluate the intubation success rate on the first attempt. The success of first intubation attempt may be affected by the experience of the operator [15]. Furthermore, Imago V-Blade® and Glidescope® have different angled blade that may slightly change the intubation maneuvers. According to these premises, we planned to not switch the anesthesiologists between the devices even if this point may introduce a bias.

## Conclusions

This feasibility study showed that the intubation with the newly introduced Imago V-Blade® is feasible. Further randomized and/or non-inferiority trials are needed to evaluate the benefit of Imago V-Blade® in this procedure.

## Abbreviations

G: Glidescope®
I: Imago V-Blade®
IDS: Intubation difficulty scale
